# Some Staff Nurses' Views

**Published:** 1918-10-12

**Authors:** 


*To the Editor of* The Hospital.

### Some Staff Nurses' Views.

*To the Editor of* The Hospital.

Sir,—May we who are chiefly concerned in this matter be allowed to give an opinion? We should like your readers to know that Major Chapple holds no authority from us to state the supposed grievance of the London Hospital nurses. If we had grievances and required public sympathy we are quite capable of asking for it without his help. As individuals we prefer to map out our own lives.

We are satisfied that the hospital authorities treat their nurses with perfect justice and according to their agreement, and we think that our opinion on this subject is more likely to be of value than that of an outsider.

As regards the efficiency of our training we think we may safely rely on the judgment of the large number of medical men who employ "London" nurses.

We have no time to enter into all the details of the controversy, but, to put it more briefly than politely, we request Dr. Chapple to mind his own business. No doubt Dr. Chapple will renew his attacks on the "London," but the public will know he is not expressing our opinions. Two Staff Nurses, on Behalf of Others,

London Hospital.

London Hospital, Whitechapel Road, E. 1.

October 5, 1918.

